# The complete chloroplast genome of *Crataegus scabrifolia* (Franch.) Rehd (Rosaceae), a medicinal and edible plant in Southwest China

**DOI:** 10.1080/23802359.2022.2160668

**Published:** 2023-01-08

**Authors:** Tian Pu, Zhen-Ning Zhao, Xiao Yu

**Affiliations:** a School of Forestry, Southwest Forestry University, Kunming, China; b School of Landscape Architecture and Horticulture Sciences, Southwest Forestry University, Kunming, China

**Keywords:** *Crataegus scabrifolia*, chloroplast genome, *Crataegus*, phylogenetic analysis

## Abstract

*Crataegus scabrifolia* (Franch.) Rehd is a medicinal and edible plant in Southwest China. The chloroplast genome of *C. scabrifolia* was analyzed by high-throughput sequencing technology, and its genetic relationship to related species was discussed. The chloroplast genome is 159,637 bp long, with two inverted repeat (IR) regions (26,384 bp each) that separate a large single-copy (LSC) region (87,730 bp) and a small single-copy (SSC) region (19,139 bp). A total of 127 genes were annotated, including 83 protein-coding genes, 8 rRNA genes, and 36 tRNA genes. The phylogenetic tree shows that *C. hupehensis* is closely related to *C. scabrifolia* with strong bootstrap support.

## Introduction

**Figure 1.**
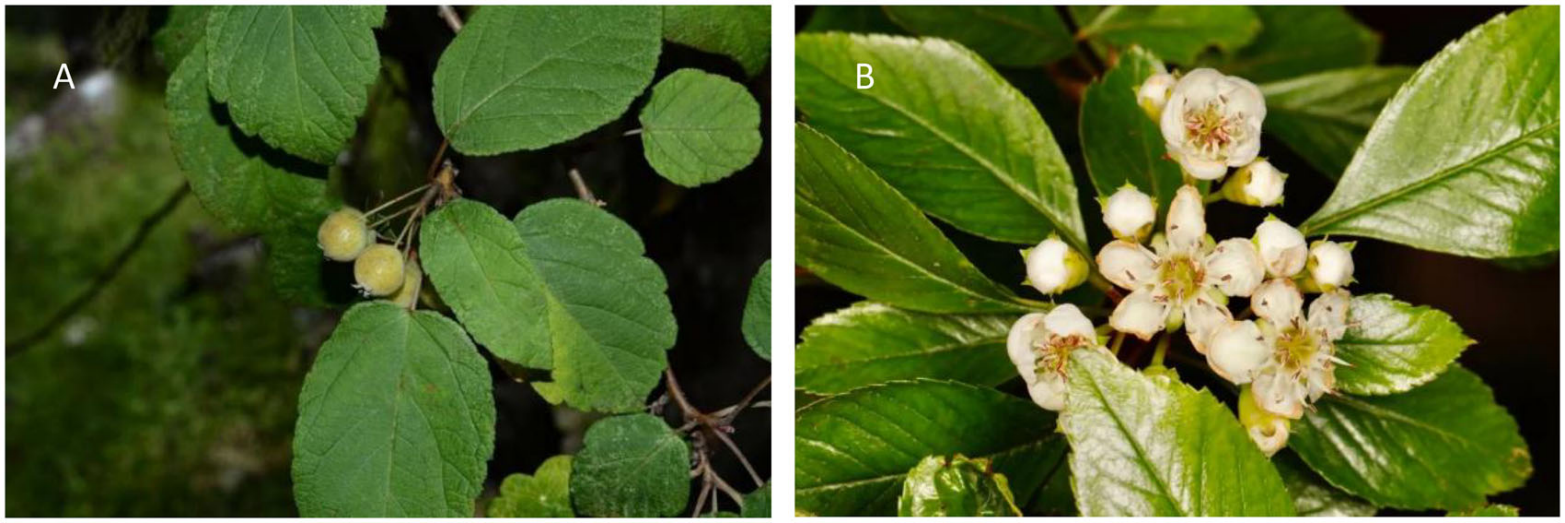
Plant morphological characteristics of *Crataegus scabrifolia*. (A) Leaves of this species ovate-lanceolate to ovate-elliptic. (B) The flowers of *C. scabrifolia* are corymbos or compound corymbos. The photos of *C. scabrifolia* were taken by the authors in Luoping Mountain, Eryuan County, Yunnan Province, China (coordinates: 99°52′19.15″E, 25°59′53.34″N).

*Crataegus scabrifolia* (Franch.) Rehd (1890) is a deciduous plant belonging to the genus *Crataegus* of the Rosaceae family (Figure 1). It is mainly distributed in the Yunnan Provinces, Guizhou Provinces, and Sichuan Provinces of China (Wu 1974). It grows in hillside mixed forests or secondary shrubs or forest margins at an altitude of 800–2400 m (Zhu et al. 2021). *Crataegus* plants contain various plant components, such as sugars and sugar alcohols, flavonoids, terpenoids, phenylpropanoids, steroids, monosteroids, sesquiterpenes, lignans, hydroxycinnamic acid, organic acids, and nitrogen compounds (Jurikova et al. 2012). It has various pharmacological uses, such as anti-hyperlipidemia, anti-hypertension, anti-oxidation, anti-inflammation, antibacterial, anti-cancer, anti-cardiac remodeling, anti-coagulation and anti-thrombus, anti-angiotensin converting enzyme, anti-arrhythmic, and anti-cataract (Martinelli et al. 2021). The medicinal parts of *C. scabrifolia* are mainly the fruit, but its pulp, leaves, and seeds can also be used as medicine (Wang et al. 2013; Kim et al. 2022;). As a medicinal and edible plant, there are few reports on the research of *C. scabrifolia* at present, which only reported its chemical constituents (Dahmer and Scott 2010). In this study, we characterized a complete chloroplast genome of *C. scabrifolia* and confirmed the phylogenetic relationship of the genus to provide an important basis for further study of the phylogenetic relationship and genetic diversity of *Crataegus*.

## Materials and methods

### DNA extraction and sequencing

Fresh leaves of *C. scabrifolia* were collected from Luoping Mountain, Eryuan County, Dali Bai Autonomous Prefecture, Yunnan Province, China (coordinates: 99°52′19.15″E, 25°59′53.34″N; altitude: 1900 m). The collection of specimens in this study did not require special permits. This research was conducted in accordance with relevant Chinese laws. A voucher specimen (SWFU20210783MFY) was deposited in the Herbarium of Southwest Forestry University, China (http://bbg.swfu.edu.cn/, Yu Xiao, email: yuxiao0215@gmail.com). Complete chloroplast DNA was extracted from dried leaf specimens of *C. scabrifolia* using the CTAB extraction method (Doyle and Doyle 1987). A total of 3 G of raw data from the Illumina Hiseq Platform (Illumina, San Diego, CA) were sequenced. Afterward, the raw data were used to assemble the complete chloroplast genome using GetOrganelle software (Jin et al. 2020) with *C. pinnatifida* (NC_057086.1) as the reference. The complete cp genome of *C. scabrifolia* was a typical quadripartite structure (Figure S1 and Figure. S2). The annotated results were modified using Geneious Prime (Kearse et al. 2012). The complete chloroplast genome of *C. scabrifolia* has been submitted to GenBank with the accession number OP021659. The OGDRAW program (https://chlorobox.mpimp-golm.mpg.de/index.html) was used to draw a detailed physical map of the *C. scabrifolia* chloroplast genome.

**Figure 2.**
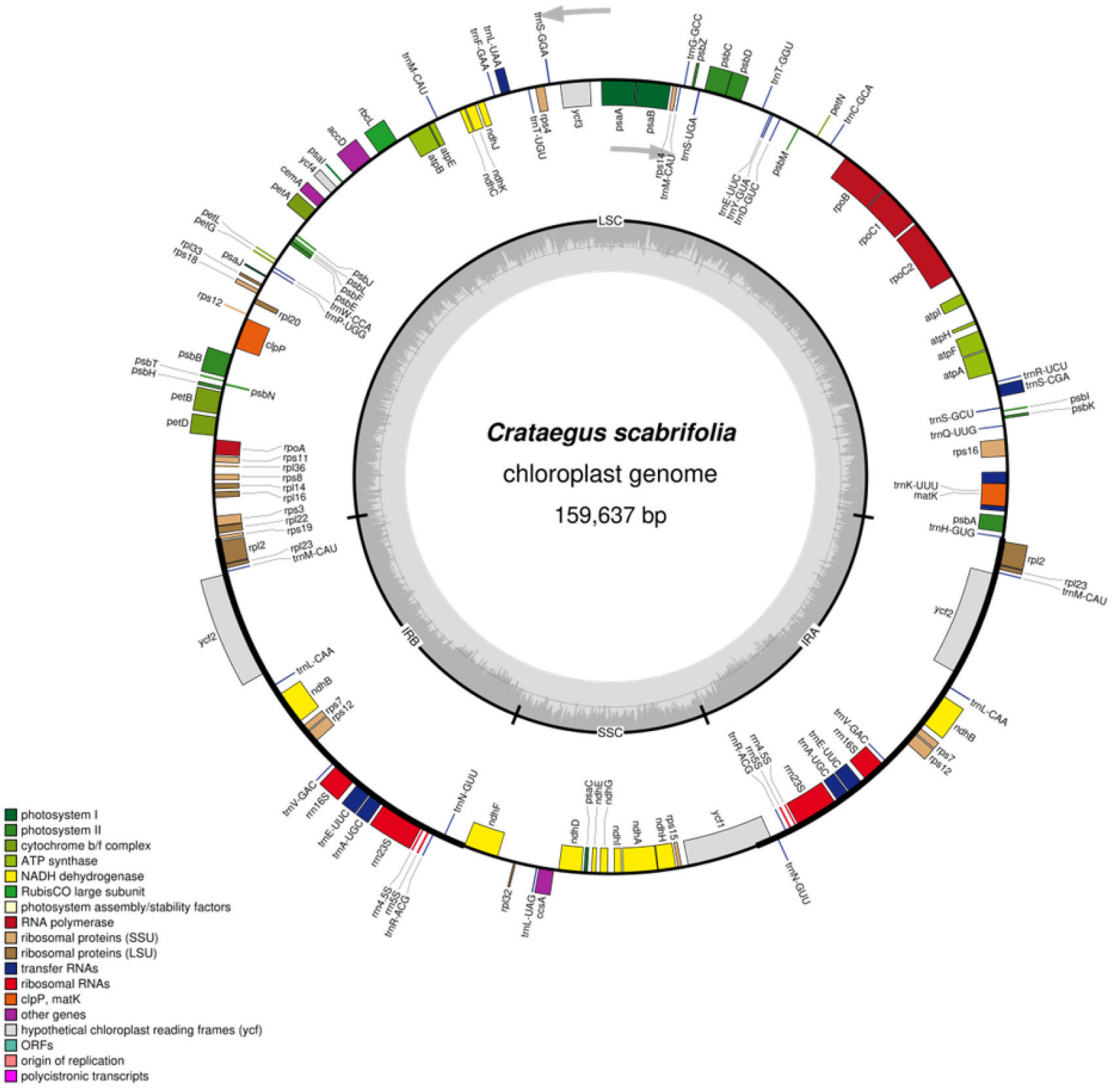
Gene map of the *Crataegus scabrifolia* plastid genome. Genes drawn inside the circle are transcribed clockwise, and those outside are transcribed counterclockwise. Genes belonging to different functional groups are color coded. The darker gray in the inner circle corresponds to DNA G + C content, while the lighter gray corresponds to A + T content. LSC: large single-copy; SSC: small single-copy; IR: inverted repeat.

### Simple sequence repeat analysis

Simple sequence repeats (SSRs) of *C. scabrifolia* were identified and localized using online MISA software (Beier et al. 2017). The repeat numbers of mononucleotide, dinucleotide, trinucleotide, tetranucleotide, pentanucleotide, and hexanucleotide were set to 10, 5, 4, 3, and 3, respectively. Identification of interspersed nuclear elements (INEs) included forward repeats, reverse repeats, palindromic repeats, and complementary repeats using the online Repter software (Kurtz et al. 2001). The maximum number of repeats was set to 50 and the minimum repeat size to 8 bp.

### Phylogenetic analysis

A phylogenetic tree was reconstructed based on the chloroplast genome of *C. scabrifolia* and 21 species of Rosaceae, with *Albizia julibrissin* (NC_058305.1) and *Cassia fistula* (ON099431.1) as the outgroup. The MAFFT software was used for multiple alignments between the chloroplast genome of these 24 plants (scoring matrix = 200; PAM *k* = 2; gap open penalty = 1.53; offset value = 0.123) (Katoh and Standley 2013); the differences between the sequences were tested. Subsequently, the alignment results were checked using MEGA11 software, and the file was output in *.NET format. Next, an ML phylogeny tree was constructed using RAxML ver. 8.0.0. The parameters were set as bootstrap = 1000 and m = GTR + GAMMA (Stamatakis 2014). The maximum-likelihood phylogenetic tree was visualized using Fig Tree 1.4.3 software (http://tree.bio.ed.ac.uk/software/figtree/).

## Results and discussion

### Structural characteristics

The complete chloroplast genome of *C. scabrifolia* is 159,637 bp in length with a typical double-stranded circular tetrad structure, containing a small single-copy (SSC) region with a length of 19,139 bp, a large single-copy (LSC) region with a length of 87,730 bp, and a pair of inverted repeat (IR) regions with a length of 26,384 bp (Figure 2). The contents of A, T, C, and G were 31.26%, 32.09%, 18.69%, and 17.96%, respectively. The overall GC content was 36.65% (LSC, 34.39%; SSC, 30.56%; IR, 42.64%). In total, 127 unique genes were annotated, including 83 protein-coding genes (PCGs), 8 ribosomal RNA genes (rRNAs), and 36 transfer RNA genes (tRNAs). Of the 127 genes encoded, there were 14 dual-copy genes, including 2 ribosome macro unit genes (*rpl2*, *rpl23*), 1 ribosome subunit gene (*rps7*), 1 NADH dehydrogenase gene (*ndhB*), 4 rRNA genes (*rrn5*, *rrn4.5*, *rrn23*, *rrn16*), 5 tRNA genes (*trnV-GAC*, *trnR-ACG*, *trnN-GUU*, *trnL-CAA*, *trnA-UGC*), and 1 unknown functional gene (*ycf2*). A total of 14 genes (*atpF*, *ndhA*, *ndhB*, *petB*, *petD*, *rps16*, *rpl2*, *rpl22*, *rpoC1*, *trnA-UGC*, *trnE-UUC*, *trnK-UUU*, *trnL-UAA*, *trnS-CGA*) contain one intron, and three genes (*ycf3*, *rps12*, *clpP*) contain two introns. The GC content in the IR region was much higher than in the LSC and SSC regions, and the different distribution of GC content was a typical feature of angiosperms (Kim and Lee 2004; Terakami et al. 2012). The main reason behind this phenomenon is that there are four GC-rich rRNA genes in the IR region (Qian et al. 2013).

### Sequence repeats analysis

A total of 98 SSRs were discovered by the online software MISA-web (Beier et al. 2017), with the numbers of mono-, di-, tri-, tetra-, pentanucleotides, and hexanucleotide SSRs being 72, 20, 1, 4, 1, and 0, respectively. According to the frequency of classified repeat types (considering sequence complementarity), mononucleotide repeats have A/T and C/G, 67 and 5, respectively; dinucleotide repeats have AG/CT and AT/AT, 1 and 19, respectively; trinucleotide repeats have only one AAT/ATT; tetranucleotide repeats have four AAAT/ATTT; and pentanucleotide repeats have only one AATCC/ATTGG. The single nucleotides in the *C. scabrifolia* chloroplast genome are biased toward A/T repeats. They have the largest number, which is in line with the results of the largest number of single nucleotides A and T in previous studies (Kuang et al. 2011; Yang et al. 2021;). A total of 50 repeats were identified in the chloroplast genome of *C. scabrifolia*, including 26 forward repeats, 7 reverse repeats, 14 palindromic repeats, and 3 complementary repeats. There are 36 repeats with a length of 20–30 bp, accounting for the majority (72%).

### Phylogenetic analyses

**Figure 3.**
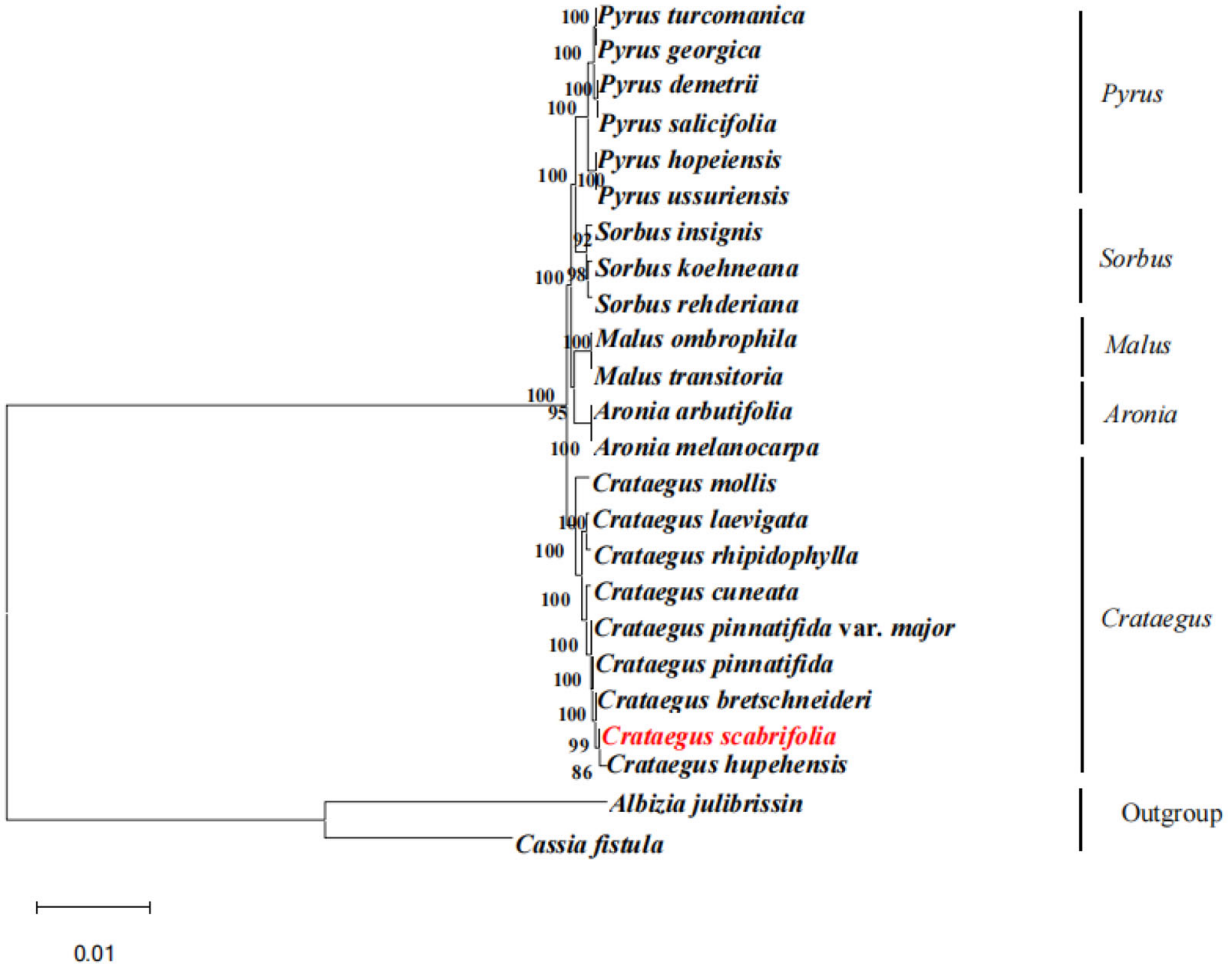
The best maximum-likelihood (ML) phylogenetic tree reconstructed by RAxML ver. 8.0.0 based on complete chloroplast genome sequences from 22 species of Rosaceae, including 9 *Crataegus* species, *Albizia julibrissin,* and *Cassia fistula* as outgroup. The numbers on branches are bootstrap support values from 1,000 replicates. The following sequences were used: *Pyrus turcomanica* NC 061559.1 (Yang et al. 2020); *Pyrus georgica* NC_061558.1 (Wang et al. 2016); *Pyrus demetrii* NC 061933.1 (Hong et al. 2021); *Pyrus salicifolia* NC 061935.1 (Katayama and Uematsu 2005); *Pyrus hopeiensis* MF521826.1 (Li et al. 2021); *Pyrus ussuriensis* NC 041461.1 (Boby et al. 2021); *Sorbus insignis* NC 051947.1 (Tan et al. 2020); *Sorbus koehneana* NC 063569.1(Li et al. 2021); *Sorbus rehderiana* OK012001.1(Ma et al. 2007); *Malus ombrophila* MW115598.1 (Lu et al. 2022); *Malus transitoria* MK098838.1 (Lu et al. 2022); *Aronia arbutifolia* NC 045391.1 (Taheri et al. 2013); *Aronia melanocarpa* MT527725.1 (Jurikova et al. 2017); *Crataegus mollis* NC 062346.1(Cha et al. 2018); *Crataegus laevigata* NC 062347.1 (Nguyen et al. 2021); *Crataegus rhipidophylla* NC 062345.1 (Żurek et al. 2021); *Crataegus cuneata* NC 058896.1 (Cui et al. 2022); *Crataegus pinnatifida* var. *major* MW653326.1 (Pang et al. 2021); *Crataegus pinnatifida* MW653325.1 (Guo et al. 2021); *Crataegus bretschneideri* MW963339.1(Zheng et al. 2021); *Crataegus hupehensis* NC 054155.1 (Hu et al. 2021); *Albizia julibrissin* NC 058305.1 (Zhang et al. 2021); *Cassia fistula* ON099431.1 (Sharma et al. 2021).

Based on the phylogenetic analysis, all species of *Crataegus* have formed a monophyletic clade. In addition, the analysis results showed that all *Crataegus* plants are divided into two clades, among which *Crataegus mollis* formed a single clade, and the other *Crataegus* plants formed a compound clade. *C. hupehensis* is closely related to *C. scabrifolia*, with a bootstrapped support rate of 86% (Figure. 3). The present sample strongly supported the taxonomic result. The fruits of the two species are red and the inflorescences are glabrous. The phylogenetic analysis results are also consistent with the classification of Flora Reipublicae Popularis Sinicae (Wu 1974). The complete chloroplast genome of *C. scabrifolia* can provide reference value and a theoretical basis for further taxonomic research, genetic engineering, and comparative genomics.

## Conclusions

The *C. scabrifolia* chloroplast genome was obtained using the Illumina HiSeq sequencing platform. The repeated sequences in this experiment may provide more specific and effective molecular markers for the classification, phylogenetic evolution, development, and gene map construction of *Crataegus* resources.

## Supplementary Material

Supplemental Material

## Data Availability

The data that newly obtained at this study are available in the NCBI under accession number of OP021659 (https://www.ncbi.nlm.nih.gov/nuccore/OP021659). The associated BioProject, SRA, and Bio-Sample numbers are PRJNA860797, SRR20339770, and SAMN29862852, respectively.
