# Supplementary material for: The complete chloroplast genome of Crataegus scabrifolia (Franch.) Rehd (Rosaceae), a medicinal and edible plant in Southwest China
Source: Mitochondrial DNA B Resour. 2023 Jan 8;8(1):81–5. doi: 10.1080/23802359.2022.2160668 (PMC9833407; doi:10.1080/23802359.2022.2160668)
Supplement: Supplemental Material [file TMDN_A_2160668_SM4039.docx]

**Content：**

**Figure S1.** The coverage figure of the complete genome

**Figure S2.** The coverage figure of the junction of the chloroplast genome

*
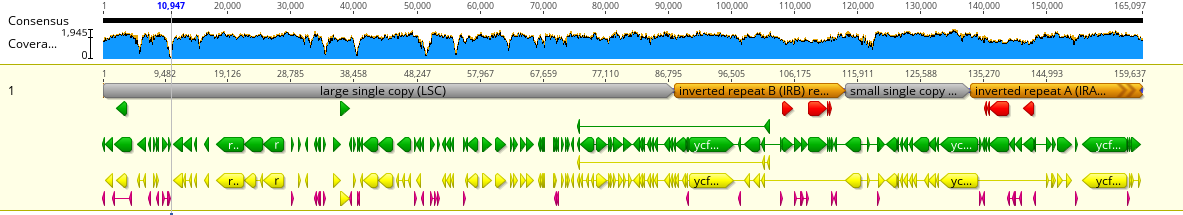
*

**Figure S1. The coverage figure of the complete genome**

*
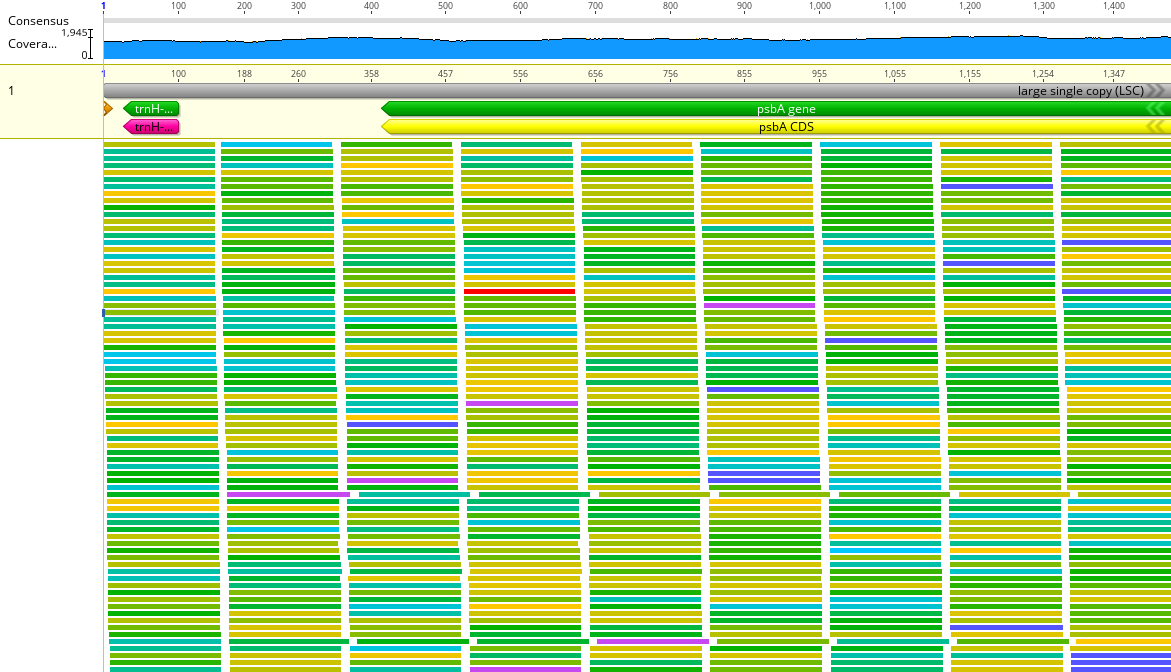
*

*
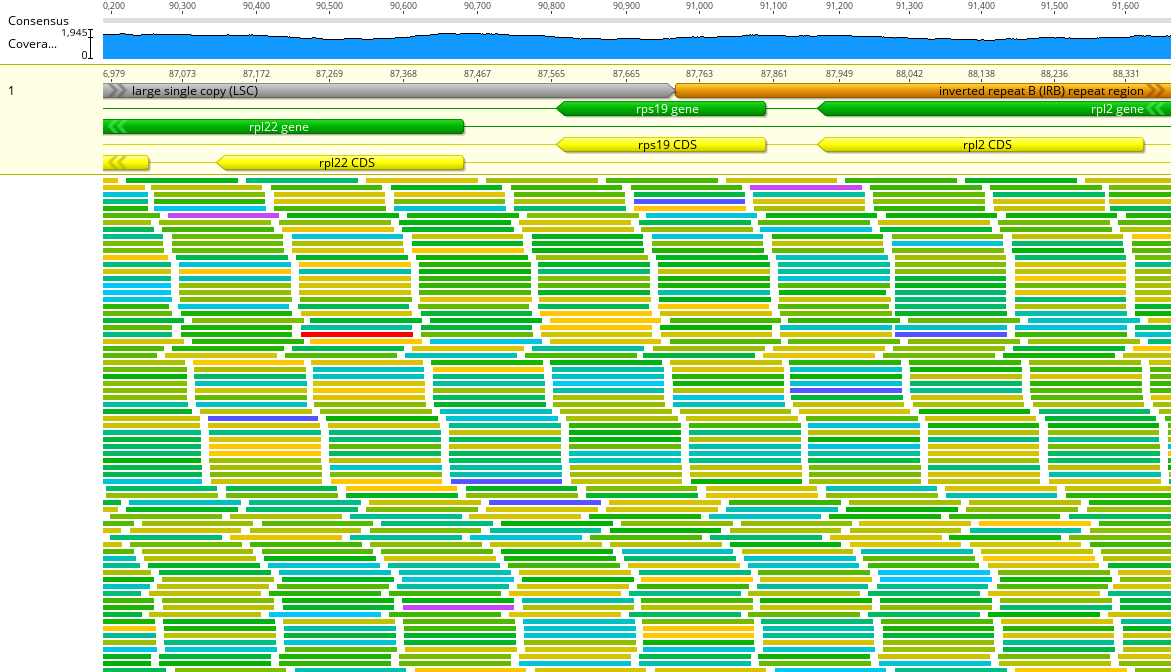
*

*
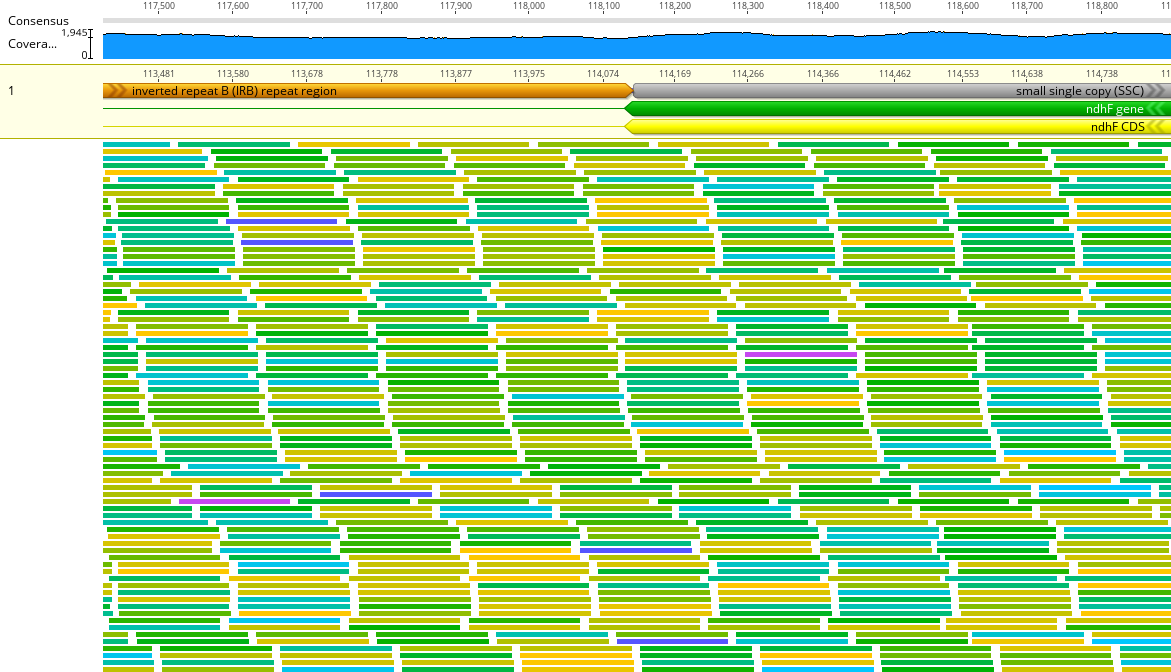
*

*
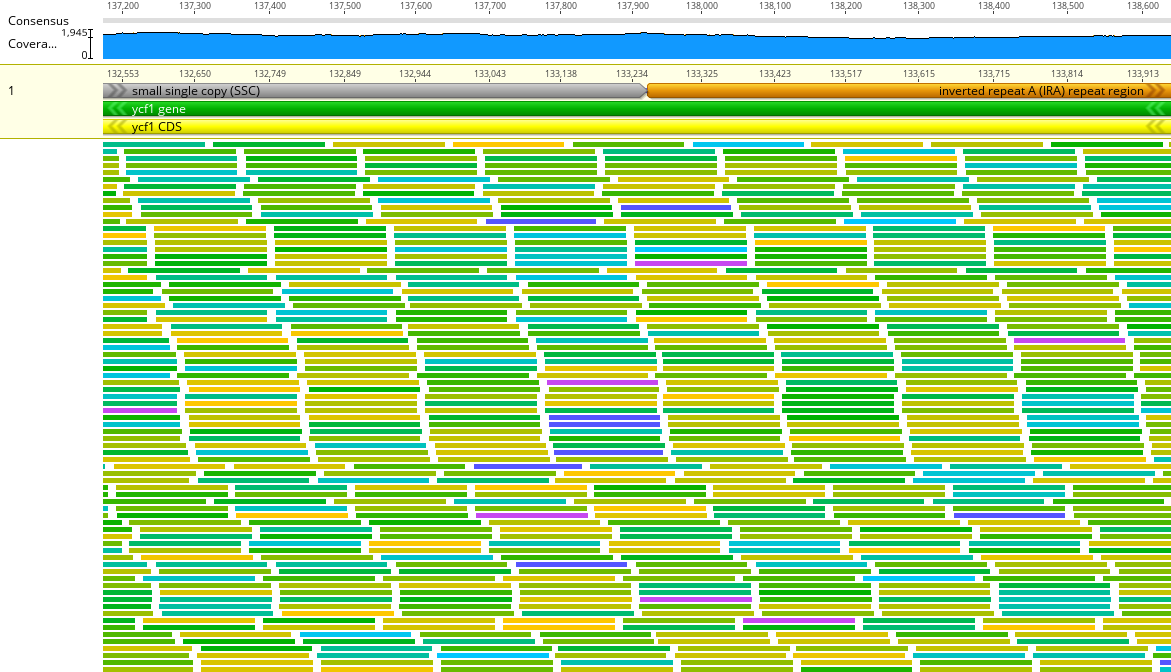
*

*
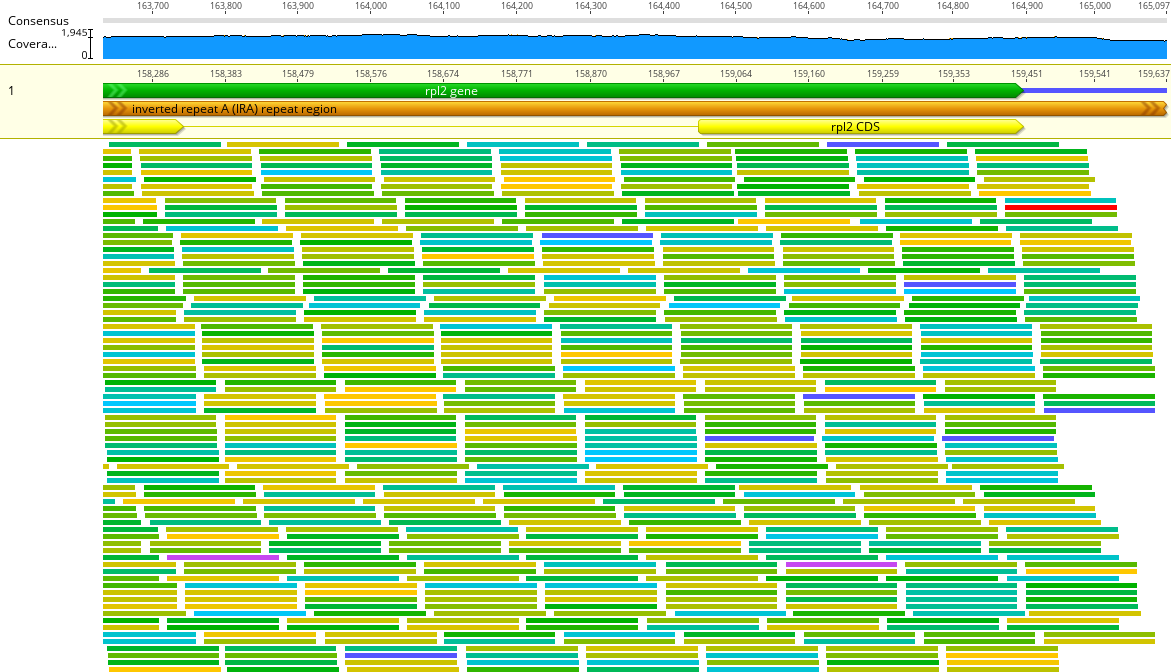
*

**Figure S2. The coverage figure of the junction of the chloroplast genome**
